# Pandemic risk-related behaviour change in England from June 2020 to March 2022: the cross-sectional REACT-1 study among over 2 million people

**DOI:** 10.1136/bmjph-2025-002851

**Published:** 2025-12-25

**Authors:** Nicholas Steyn, Marc Chadeau-Hyam, Matthew Whitaker, Christina Atchison, Deborah Ashby, Graham S Cooke, Helen Ward, Paul Elliott, Christl A Donnelly

**Affiliations:** 1 Department of Statistics, University of Oxford, Oxford, UK; 2 MRC Centre for Environment and Health, Imperial College London, London, UK; 3 School of Public Health, Imperial College London, London, UK; 4 Faculty of Medicine, Imperial College London–South Kensington Campus, London, UK; 5 Department of Infectious Disease, NIHR Imperial Biomedical Research Centre, London, UK; 6 Imperial College Healthcare NHS Trust, London, UK; 7 Department of Infectious Disease, Imperial College London, London, UK; 8 NIHR Imperial Biomedical Research Centre, London, UK; 9 MRC Centre for Environment and Health, London, UK; 10 Pandemic Sciences Institute, University of Oxford, Oxford, UK

**Keywords:** COVID-19, Epidemics, Humans, Population Surveillance, Social Cohesion

## Abstract

**Objective:**

To determine how people in England changed their infection risk-related behaviours during the COVID-19 pandemic and in response to control measures, 19 June 2020 to 31 March 2022.

**Design:**

Over 18 (of 19) rounds, randomly selected participants across England completed a questionnaire about risk-related behaviours, sociodemographics and symptoms.

**Participants:**

Between 85 018 and 154 060 randomly selected participants per round, aged 5+ years, totalling 2 177 657 responses with relevant data.

**Main outcome measures:**

Primary outcomes were self-reported shielding and/or taking specific precautions, not leaving home in the prior week, not being in close proximity with anyone outside their household the day before and wearing face coverings outside the home. Secondary community-level measures of mobility and public health policy stringency were compared with the primary outcomes to provide population-level context to the observed findings.

**Results:**

Infection risk-related behaviours varied considerably over the nearly 2 years under study. Protective behaviours peaked in January 2021, during England’s winter wave, before widespread vaccination. At that time, the estimated proportion of self-reported shielding and/or taking specific precautions reached 21.6% (95% CI 21.4% to 21.8%), of self-reported not leaving home in the week prior to completing the questionnaire reached 7.99% (95% CI 7.85% to 8.13%) and of self-reported not having contact with anyone outside their household on the day before answering the questionnaire reached 89.2% (95% CI 89.1% to 89.4%). As self-reported vaccination rates increased and prevalence of infection decreased, protective behaviours decreased, although patterns varied by demographics. Protective behaviours were strongly correlated with community-level mobility data and the stringency of public health measures.

**Conclusions:**

Individual-based data showed sizeable proportions of people undertook protective behaviours during the pandemic, especially during the second lockdown in January 2021, although there was evidence of ‘pandemic fatigue’ in the study’s later stages. Self-reported behaviours were closely aligned with community mobility data and the stringency of government policies, indicating policy-driven behavioural changes.

**WHAT IS ALREADY KNOWN ON THIS TOPIC**

- Behavioural changes in response to infectious disease threats and public health interventions are known to influence disease transmission.

**WHAT THIS STUDY ADDS**

- Precise data on the willingness and ability of populations, such as those in England, to change their behaviours in response to infectious disease threats are scarce and challenging to collect. In this study, we use repeated large-sample representative surveys to show that behavioural responses in England during the COVID-19 pandemic closely aligned with the timing of government-imposed restrictions.

**HOW THIS STUDY MIGHT AFFECT RESEARCH, PRACTICE OR POLICY**

- This study offers detailed insights into how demographics and characteristics such as vaccination status and prior infection impacted risk-related behaviours during COVID-19, providing valuable insights for future outbreak response planning. We also use our data to validate aggregated population-level data sources, such as the Google community mobility series, as proxies for population mobility—justifying their use in a range of epidemic modelling contexts.

## Introduction

 The COVID-19 pandemic in England was a complex and quickly evolving public health crisis consisting of multiple waves of infection. The REal-time Assessment of Community Transmission-1 (REACT-1) study provided real-time estimates of the prevalence of SARS-CoV-2 swab positivity in England from 1 May 2020 to 31 March 2022.^1^ Conducted over 19 rounds, more than 2.5 million nasal swab samples were processed using reverse transcriptase PCR, and participants completed an extensive questionnaire about their behaviours related to infection risk, sociodemographic characteristics and symptoms.

Various non-pharmaceutical interventions (NPIs) were used throughout the pandemic, including stay-at-home orders (‘lockdowns’) on multiple occasions, notably in March 2020 (prior to the start of REACT-1),^2^ and then in the winter of 2020/2021 (around the time of study rounds 7–9).^3^ These interventions were mostly lifted in the middle of 2021, although some intermediate measures, such as recommended working from home, were reintroduced in December 2021 to slow the spread of the Omicron variant.^4^

Changes in individual behaviours, influenced by perceived risks, governmental guidelines, societal norms and ‘pandemic fatigue’, could directly impact the trajectory of the epidemic and the effectiveness of policy decisions. Understanding how these behaviours changed over the course of the pandemic is critical to quantify the impact of NPIs, to gauge their effectiveness at reducing SARS-CoV-2 transmission and to understand how compliance varied across demographic groups and over time.^5^

Community-level mobility data, such as the Google community mobility reports,^6^ have been widely used in epidemiological studies to evaluate the effectiveness of social distancing measures^7^ or to predict the rate of virus transmission.^8^ These data offer valuable insights but are only aggregated proxies for individual behaviours and for social interaction.

The Oxford COVID-19 Government Response Tracker (OxCGRT)^9^ collected information on a variety of policy measures that governments used during the pandemic, such as school closures and workplace restrictions. In particular, their stringency index has been used for comparing the strictness of policy responses between countries and has featured directly in epidemiological models.^10^ Their containment and health index closely resembles the stringency index, with additional consideration given to contact tracing, testing, face covering policy, vaccinations and protection of elderly people.

Focusing on self-reported behaviours relating to shielding and/or taking specific precautions, not leaving the home, not having physical contact/close proximity with people outside the household and wearing face coverings outside the home, we estimated the self-reported prevalence of these behaviours from 19 June 2020 to 31 March 2022 in England and by sociodemographic group. We also investigated the consistency of individually reported and community-level behavioural data by relating these estimates to (1) (Google) mobility data and (2) the OxCGRT stringency index and OxCGRT containment and health index.

## Materials and methods

### REACT-1 data

The REACT-1 study conducted 19 rounds of sampling from 1 May 2020 to 31 March 2022 with 85 018–154 060 participants per round. Participants completed a comprehensive survey reporting their risk-related behaviours, sociodemographic characteristics, health and health history, history of COVID-19 and risk tolerances. The survey was completed by the respondent (if over the age of 18 years), by the respondent’s parent/guardian (for ages 5–12) and either by the parent/guardian or by the respondent themselves (for ages 13–17). Round-specific random iterative method (RIM) weights^11^ were calculated at the time of survey administration to enable prevalence estimates that are representative of the population of England. These were calibrated for participants’ age, sex, area-level deciles of deprivation,^12^ lower tier local authority counts and ethnicity.^1^ A Public Advisory Panel provided input into the design, conduct and dissemination of the REACT research programme.

Of the questions on behaviours, we focused on one related to shielding (“*Are you shielding and/or taking specific precautions because you are concerned that you/your child will become severely ill with COVID-19?*”), one to leaving the home (“*Did you/your child leave home for any reason in the last 7 days?”* and later “*In the last 7 days, for what reasons have you left home? Select all that apply.*”), one to social distancing (“*Not including members of your household, how many different people did you have contact with yesterday? By contact we mean: any direct skin-to-skin physical contact (eg, kiss/embrace/handshake), being less than 2 metres from another person for over 5 minutes*”) and one to wearing face coverings outside home (“*Do you/does your child mainly wear any kind of face covering or mask when you/they are outside your/their home, because of COVID-19?*”). The specific question wording occasionally changed between study rounds (online supplemental section 1.1: online supplemental tables 1–3).

During study round 1, the relevant questions were not included in the questionnaire; we therefore excluded the round 1 participants from this analysis (n=120 620). Of the REACT-1 participants in study rounds 2–19 (19 June 2020 to 31 March 2022) (n=3 239 620), we excluded, prior to analysis, participants with invalid survey weights (n=1019) as well as those who registered but did not complete the questionnaire (n=1 060 944). Some participants did not answer every question, provided an invalid response or were not asked a specific question (the latter primarily being due to age). When responses to individual questions were missing, the response was discarded (set as ‘NA’). Hence, reported proportions are relative to the total number of respondents to each question. A round-by-round breakdown of non-response rates by question is provided in online supplemental section 1.2: online supplemental tables 5 and 6.

### Google community mobility

Community mobility data were obtained from Google for the period 15 February 2020 to 15 October 2022.^6^ These publicly available data were aggregated from device users who had turned on the location history setting (by default it is off). These data present the day-to-day percentage change (relative to a day-of-the-week-specific baseline calculated from the 5-week period from 3 January 2020 to 6 February 2020) in the total number of visitors to places of retail and recreation, grocery and pharmacy, parks, transit stations and workplaces, as well as the daily percentage change in time spent at residential locations.

### Oxford COVID-19 Government Response Tracker

The OxCGRT stringency index and containment and health index were obtained for the period 1 January 2020 to 31 December 2022. These data aggregate indicators measuring the implementation of school closures, workplace closures, public event cancellations, restrictions on gathering sizes, closure of public transport, stay-at-home requirements, restrictions on internal movement, restrictions on international travel and public health information campaigns. The containment and health index additionally considers testing, contact tracing, face covering requirements and vaccination policy.

### Statistical methods

Results are presented at the national level, as well as for four age groups in years (5–17, 18–34, 35–64, 65 and over) and four household sizes (lives alone, 2 people, 3–4 people, 5+ people). Additionally, we considered geographical area, area-based deciles of deprivation,^12^ ethnicity and sex^1^ in online supplemental sections 2.1–2.5. A choropleth showing aggregated area-based deciles of deprivation is also provided in online supplemental section 2.6: online supplemental figure 17.

The *svyciprop* function with the logit method, from the *survey* package in R, was used to estimate weighted round-level binomial proportions and corresponding CIs^13^ from the REACT-1 survey data. Daily prevalence estimates were unweighted proportions because day-specific RIM weights were not available, and the corresponding CIs were calculated using the *binconf* function from the *Hmisc* package in R.^14^ The RIM weights account for response bias associated with individual demographics but not for response bias independently associated with the outcome of interest (eg, if those who report shielding are more likely to respond to the survey). We demonstrate a calculation to adjust for hypothetical levels of this bias in online supplemental section 2.7: online supplemental figure 18.

We analysed if and to what extent responses to the behavioural questions varied by age group, sex, geographical area, deprivation, ethnicity and household size, via survey-weighted logistic regression models. These models were fit to data from each study round using the *svyglm* function with the binomial family from the *survey* package in R.^13^ Results from these models are presented as ORs, providing the relative odds of reporting a given behaviour compared with a specified reference group.

To evaluate the consistency of the Google community mobility data and the OxCGRT indices with self-reported behaviours, we fitted random forest regression models, using daily aggregated REACT-1 data to predict each mobility series and index separately. When the mobility series was used as the outcome variable, we used the daily proportion of people leaving their homes for various reasons (and not leaving) as predictors (the variables are listed in online supplemental table 3). When the OxCGRT indices were used as the outcome variable, we used the daily proportion of people reporting shielding and/or taking specific precautions, not leaving the home in the prior week, not having physical contact/close proximity with people outside their household the day before and wearing face coverings outside the home as predictors (four in total). To reduce the impact of day-of-the-week variability in the mobility data, we smoothed these series using a trailing 7-day moving average prior to fitting the models. To limit measurement error in our predictors, we only considered days when more than 200 survey responses were recorded.

We used the *randomForest* package in R^15^ with default hyperparameters (500 trees, each fit with approximately 63.2% of the available observations, selected at random) to fit the random forest models. Model performance was assessed using the proportion of the within-bag variance explained (the proportion of variance in the response that is explained by the predictors when predicting data used to fit the models) and out-of-bag variance explained (the proportion of variance in the response that is explained when predicting data not used to fit the models). The within-bag variance explained is analogous to R-squared in a linear regression context.

Three separate models for each Google community mobility series were constructed to reflect changes in question formulation between study rounds. We defined three main periods (1) from 19 June 2020 to 11 November 2020 (study rounds 2–6, n=94 days with >200 survey question responses, 11 predictors), (2) 4 January 2021 to 28 September 2021 (study rounds 8–14, n=135 days with >200 survey question responses, 8 predictors) and (3) 19 October 2021 to 31 March 2022 (study rounds 15–19, n=104 days with >200 survey question responses, 14 predictors). Study round 7 was not included in this analysis as the question regarding reasons for leaving the home in the past 7 days was not asked. One model was fit for each OxCGRT index, covering the period 16 October 2020 to 31 March 2022 (study rounds 6 and 8–19, n=259 days with >200 survey question responses, 4 predictors).

### Data portal

We have developed an online portal to facilitate visualisation and download of aggregated behavioural data while protecting the confidentiality of survey participants. This can be accessed here: https://m-whit-ic.shinyapps.io/react-social-shiny/. Further information about this portal is provided in online supplemental section 6.

### Patient and public involvement

A REACT Public Advisory Group (PAG) was established in early 2020 (see https://www.imperial.ac.uk/medicine/research-and-impact/groups/react-study/react_pag/) and met regularly to shape the research and provide input into key aspects of design, delivery, interpretation and dissemination. The PAG was involved in discussions on the wording of invitations and use of incentives^16^ to improve uptake and specifically inclusion of groups with lower rates of participation. The PAG receives regular updates on the conduct of the study including changes to survey questions, proposed analyses and preliminary results. PAG contributions have included discussion of the design of the at-home testing programme, review of study materials including survey design and participant communications, coauthoring relevant publications and reports and providing ongoing strategic direction to the research to ensure public and participant perspectives inform plans at all levels. The PAG is represented on the REACT Data Access Committee. Since 2022, the PAG has expanded to include people with lived experience of long covid. Published papers are made available to participants on the study website, and regular study updates are shared in the form of infographics with the PAG and a wider community network of hundreds of people who have signed up for updates.

We acknowledge the value contribution of members of the PAG (Amara Lalji, Joan Bedlington, Maisie McKenzie, Marney Williams, Philippa Russell, Sandra Jayacodi and Steve Gibbard) to the design and conduct of the REACT study, and the Imperial Patient Experience Research Centre for establishing and facilitating this public involvement.

## Results

### Behaviours over time

**Figure 1:**
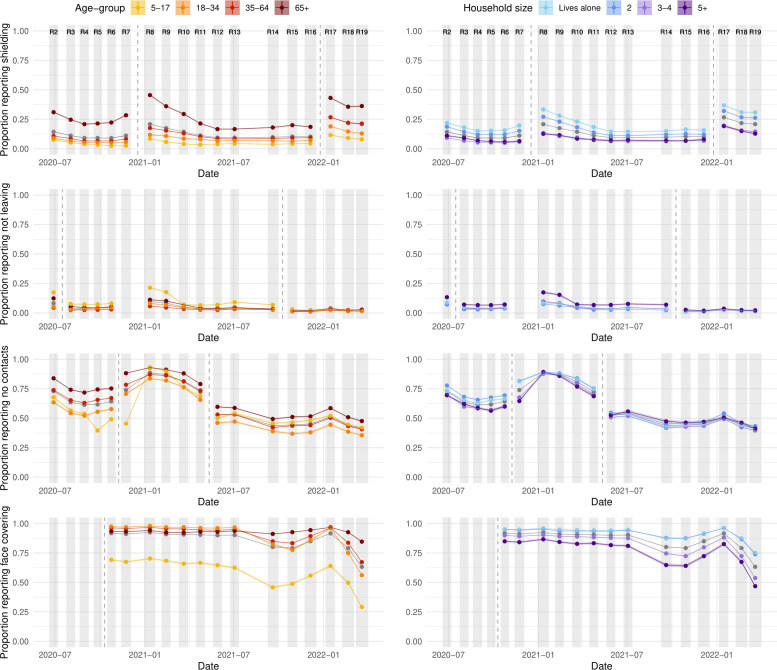
The proportion of people in England self-reporting shielding and/or taking specific precautions because they consider themselves ‘to be at risk of severe illness for COVID-19’ (first row), not leaving the home on the day preceding answering the questionnaire (second row), having no contacts outside the home in the preceding 7 days (third row) and wearing a face covering outside the home (fourth row) by age group (left column) and household size (right column). Changes in question wording are demarcated with a vertical dashed grey line—caution should be exercised in comparing values on either side. Shaded regions and vertical coloured lines show 95% CIs about the estimated proportion, although they are frequently too small to discern. Individual points are joined by lines and shading for clarity, but this should not be interpreted as an interpolation. National averages are shown in grey.

We observed a peak in reported protective behaviours in January 2021 (study round 8), at the time of the third national lockdown in England (figure 1). At this time, self-reported shielding and/or taking specific precautions reached 20.8% (95% CI 20.6% to 21.1%), self-reported not leaving home in the 7 days prior to completing the questionnaire reached 9.7% (95% CI 9.5% to 9.9%) and self-reported not having physical contact/close proximity with anyone outside the household on the day before answering the questionnaire reached 93.3% (95% CI 92.8% to 93.7%).

Following this peak, reported protective behaviours decreased over the summer months in 2021, with a noticeable uptick again in January 2022 (figure 1, study round 17), during the first Omicron wave and the government’s ‘Plan-B’ interventions and messaging.^17^ This uptick did not result in social distancing behaviours reaching the same level as winter 2020/2021, except for the proportion of people reporting shielding and/or taking specific precautions. To some extent, the increase between December 2021 and January 2022 (study rounds 16 and 17) of this behaviour can likely be attributed to rewording of the question (specifically the removal of the word ‘shielding’, a term with a much narrower definition than ‘specific precautions’). These wording changes are described in online supplemental section 1.1.

After the uptick in December 2021/January 2022, protective behaviours decreased rapidly over February and March 2022 (study rounds 18 and 19),^18^ despite infection prevalence reaching the highest measured by the study in March 2022 (weighted swab positivity in round 19 was 6.37%). The proportion self-reporting wearing face coverings reached the lowest recorded level in the study of 63.2% (95% CI 62.9% to 63.6%), down from 91.6% (95% CI 91.4% to 91.8%) in January 2022 (study round 17) and 92.4% (95% CI 92.2% to 92.6%) in January 2021 (study round 8). Similarly, we observed reductions in the proportion self-reporting no contacts outside the home (41.2%, 95% CI 40.9% to 41.6%) in March 2022, down from 51.3% (95% CI 50.9% to 51.7%) in January 2022 and 88.6% (95% CI 88.3% to 88.8%) in January 2021.

Figure 1 shows that those aged 65 years and older were more likely to report protective behaviours to limit the risk of SARS-CoV-2 infection (except for reporting not leaving home) than younger participants. A similar but less marked difference was observed for those reporting living alone when asked whether they had left the home. Responses to these behavioural questions disaggregated by other demographics are considered in online supplemental section 2: online supplemental figures 1–4—those living in neighbourhoods with greater socioeconomic deprivation were more likely to report protective behaviours, although less likely to report wearing face coverings. Results were less consistent between questions and over time for region, sex and ethnicity.

**Figure 2:**
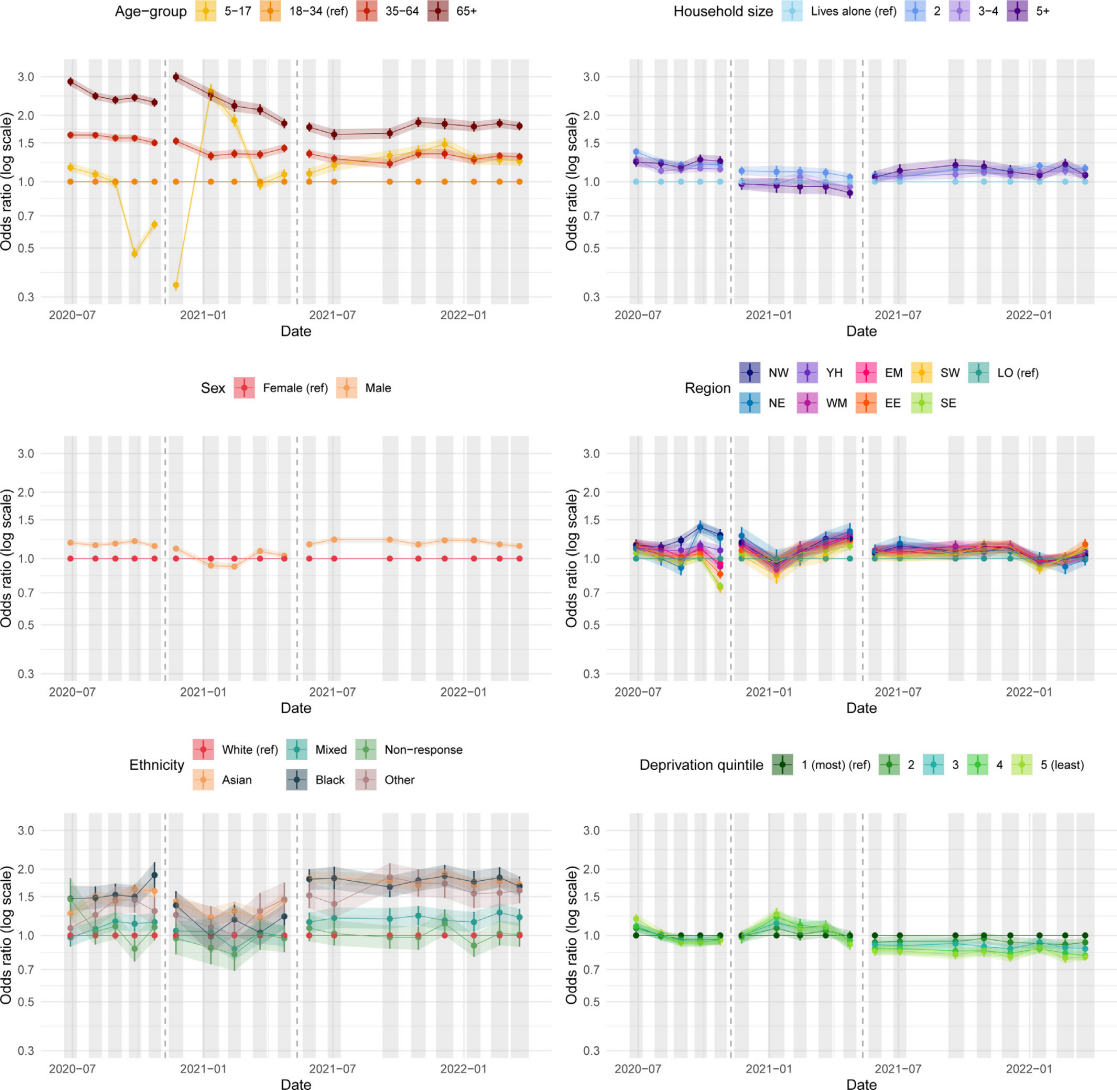
ORs for whether an individual self-reports having no contacts outside the home in the preceding 7 days. Estimates are controlled for the effect of all six demographic variables. Reference groups are indicated with (ref) in the figure legends and by a horizontal line at an OR of 1.0 in the plots. Changes in question wording are demarcated with a vertical dashed grey line—caution should be exercised in comparing values on either side. Shaded regions and vertical coloured lines show 95% CIs about the estimated OR. Individual points are joined by lines and shading for clarity, but this should not be interpreted as an interpolation. The values visualised here are reported in online supplemental figures 19–30. EE, East of England; EM, East Midlands, LO, London; NE, North East; NW, North West; SE, South East; SW, South West; WM, West Midlands; YH, Yorkshire and the Humber.

Survey-weighted logistic regression models estimating the independent effect of each demographic variable show that, compared with 18–34 year-olds, those aged 65+ were more likely to report having no contacts outside the home, with ORs between 1.64 (95% CI 1.55 to 1.73) and 2.99 (95% CI 2.84 to 3.14, p<0.05) (figure 2). The impact of 5–17 year-olds returning to school between September and November 2020 (study rounds 5–7) is also clearly visible (figure 2); compared with 18–34 year-olds, the ORs were between 0.34 (95% CI 0.32 to 0.35) and 0.64 (95% CI 0.61 to 0.67) for having no contacts outside the home over this period. These ORs were largely unaffected by further adjustment for being a self-reported suspected or confirmed COVID-19 case in the preceding 2 weeks (online supplemental section 3).

Figure 2 also suggests that men were more likely than women to report having no contacts outside the home, except in January and February 2021, while those living with other people were more likely to report having no contacts outside the home, except between November 2020 and April 2021. Further results regarding the average total number of contacts individuals had outside their home are provided in online supplemental section 2.2: online supplemental figure 5. Results from logistic regression models on additional outcomes, including specific reasons for leaving the home, are included in online supplemental section 3: online supplemental figures 19–30. The same results after controlling for infection status are reported in online supplemental figures 31–42.

**Figure 3:**
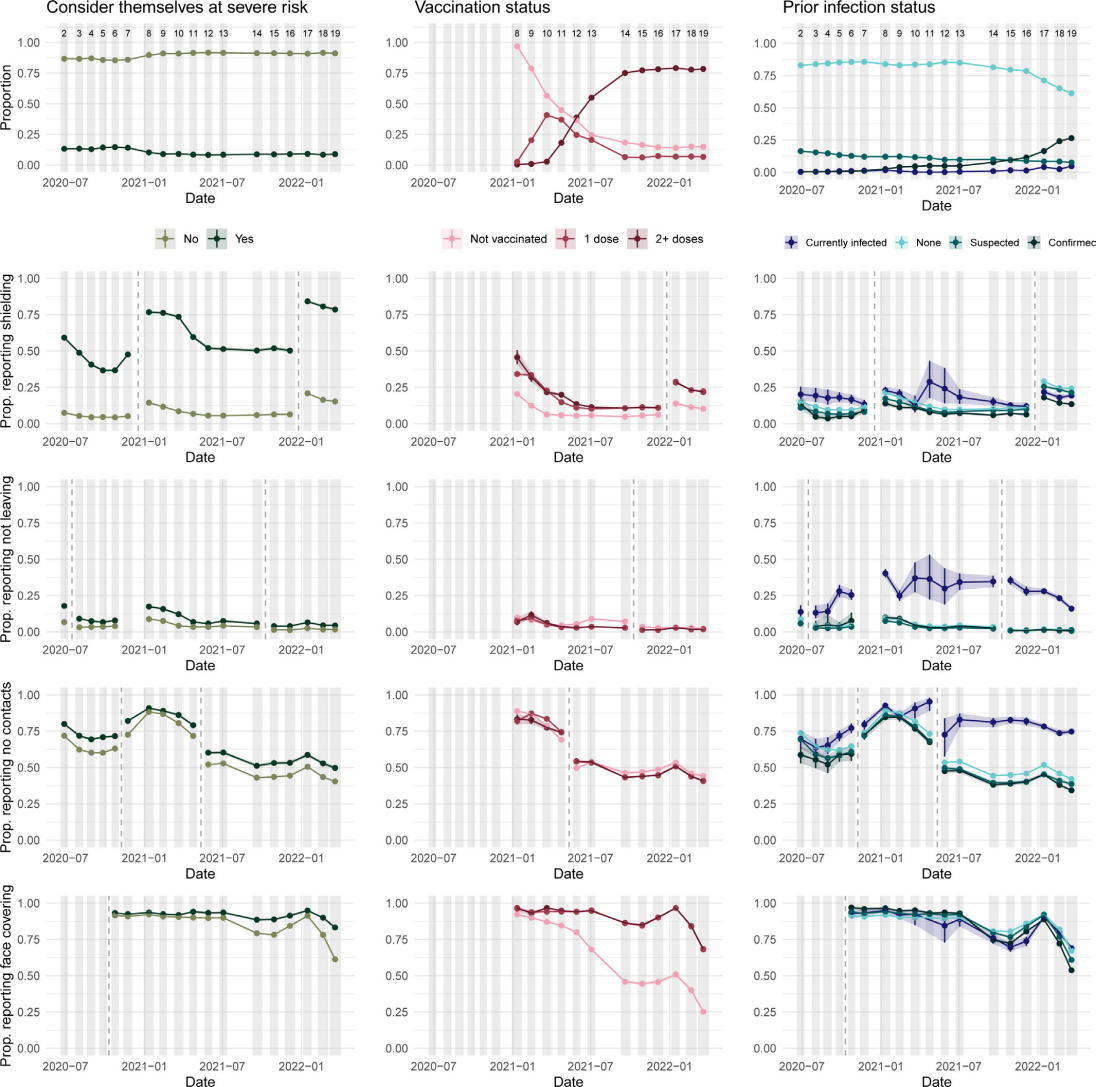
The proportion of people in England self-reporting considering themselves at severe risk, having no/one/two plus doses of a vaccine and having no/a suspected/a confirmed prior infection or current infection (top row). Rows 2–5 then present responses to the four focus questions, disaggregated by these responses. Vertical dashed lines denote changes in question wording. Study round numbers are provided at the top of row 1 and shaded regions indicate periods when the study was actively collecting data.

We also consider responses to these behavioural questions conditional on self-reported risk, vaccination status and infection history (figure 3). Those who considered themselves to be at risk of severe illness were more likely to report shielding, as were those who reported having been vaccinated, and those who had not reported previous infection. When the question was first asked, there was high self-reported use of face coverings, although over time those who did not consider themselves at severe risk and those who reported no vaccination became relatively less likely to report wearing a face covering. The proportion of people reporting being at risk, being vaccinated and having a past infection is reported in online supplemental section 2.3: online supplemental figures 6–11. Further behavioural analyses, stratified by these characteristics, are then presented in online supplemental section 2.4: online supplemental figures 12–15. The online material also provides a range of outputs conditional on self-reported risk, vaccination status and infection history (https://m-whit-ic.shinyapps.io/react-social-shiny/).

### Comparisons with mobility data

**Figure 4:**
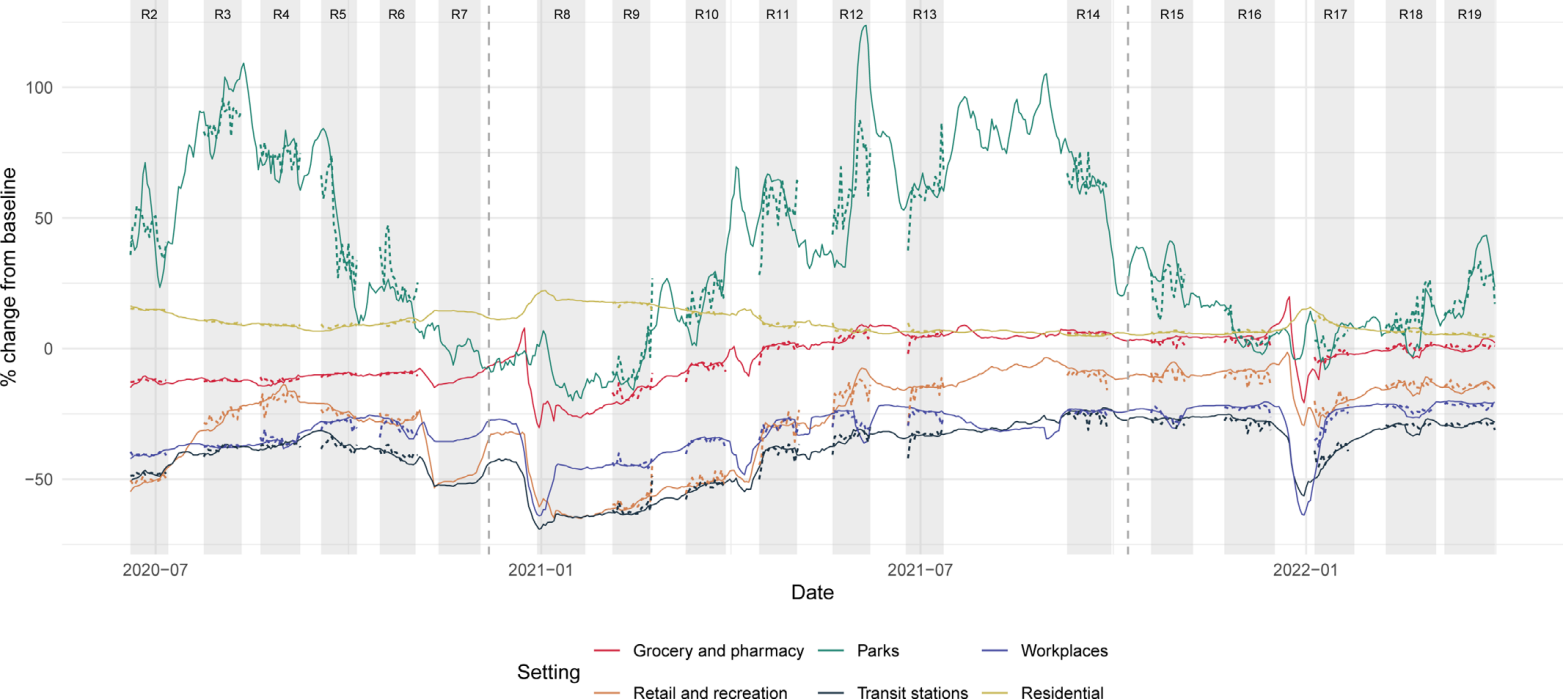
The Google mobility data (solid lines) between June 2020 and March 2022 and the random forest model predictions based on behaviours reported in REACT-1 questionnaires (dotted lines). Modelled values shown are out-of-bag predictions, so the model has not been trained on the data point being predicted. The vertical dashed lines demarcate individual model fits, selected to account for substantial changes in question wording. We do not fit a model to data from study round 7 as the relevant questions were not asked. REACT-1, REal-time Assessment of Community Transmission-1.

Despite the limited duration of each period considered (19 June 2020 to 11 November 2020; 4 January 2021 to 28 September 2021; and 19 October 2021 to 31 March 2022), substantial variation in mobility and behaviours was observed within each period. The random forest models of the mobility series capture key trends while missing some finer day-to-day variations (figure 4). The out-of-bag proportion of variance explained (PVE) (online supplemental table 8) was high for all six mobility indices (ranging from 86% to 97%) for data from study rounds 8–14 and was lower for study rounds 15–19 (ranging from 68% to 86%). Additional results show that shopping was consistently the main reason for leaving the home, while the proportion of individuals reporting errands and work as a reason for leaving the home increased from January 2021 onwards (online supplemental section 2.5: online supplemental figure 16). Correlation coefficients between the self-reported behaviours and mobility series are reported in online supplemental section 4: online supplemental table 7.

### Comparisons with policy response indices

Results from the random forest models for the two policy response indices demonstrate a strong association between self-reported behavioural data and the stringency indices. The out-of-bag PVE was 0.98 for both models demonstrating a strong association between self-reported behaviours and aggregated measures of the stringency of public health policy.

**Figure 5:**
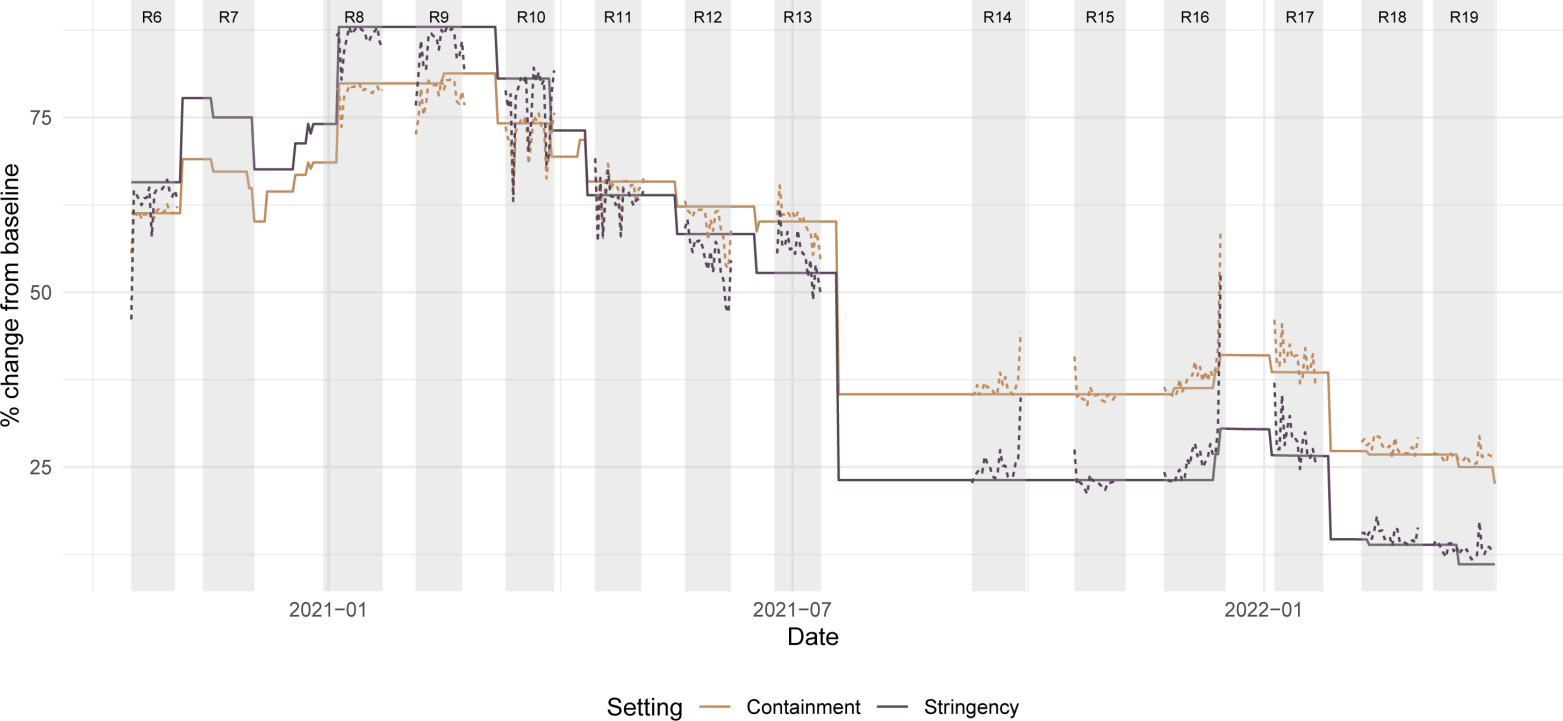
The OxCGRT stringency index and containment and health index (solid lines) between October 2020 and March 2022 and the model predictions based on behaviours reported in REACT-1 questionnaires (dotted lines). We do not fit the model to data from study round 7 as the relevant question was not asked in this round. OxCGRT, Oxford COVID-19 Government Response Tracker; REACT-1, REal-time Assessment of Community Transmission-1.

The fitted index values based on behavioural data also identified the timing of when the change from baseline in the stringency index switched from being above to below that for the containment and health index, which occurred near the start of study round 11 (figure 5).

For comparison, we also fit a logistic regression model to the policy indices using the Google mobility data as the predictors (online supplemental section 5: online supplemental figure 43). Responses to the four risk-related behavioural questions from the REACT-1 study explained more variance in the policy index than did the six Google mobility variates (online supplemental table 8), with an out-of-bag PVE (using the Google data) of 0.91 for the stringency index and 0.90 for the containment and health index (online supplemental table 9) (compared with the out-of-bag PVE of 0.99 for the model leveraging REACT-1 data), despite the Google mobility series using more covariates.

## Discussion

The COVID-19 pandemic was a major shock to health systems requiring near-unprecedented responses from governments and individuals to protect individual and public health. Government response in England and many other countries included imposition of lockdowns and other containment measures, with legal restrictions on population mobility and behaviours. We show strong evidence that individuals substantially changed their behaviours over time, with behaviours to reduce risk of infection closely reflecting these policy shifts during the pandemic.

Within the period considered (19 June 2020 to 31 March 2022), protective behaviours were most pronounced in January 2021, during the winter wave of COVID-19 in England, when very few people had been vaccinated. Over the following months, vaccination rates increased while the prevalence of infection decreased, and the proportion of people who reported protective behaviours decreased.

Infection prevalence sharply increased over December 2021 and January 2022 due to the Omicron BA.1 variant^4^ accompanied by an uptick in protective behaviours, although not to the previous levels seen in January 2021 during the second national lockdown. This was then followed by a second wave during February and March 2022 due to the Omicron BA.2 variant, resulting in the highest recorded SARS-CoV-2 prevalence in REACT.^18^ However, at the same time, there was a fall in protective behaviours, indicating possible ‘pandemic fatigue’. This is particularly noticeable in the wearing of face coverings, which decreased rapidly over February and March 2022 to an all-time low, after being at relatively constant levels since face covering wearing was first recommended in July 2020.

We found important differences between demographic groups. For example, those living in larger households were generally less likely to report shielding, but were more likely to report not leaving the home. There were also large differences by age, and at times, by sex, ethnicity and region. In particular, we found that women were consistently more likely to report wearing a face covering outside the home than men (online supplemental figure 4), reflecting wider trends observed during the pandemic in other regions.^19 20^ The OR associated with wearing a face covering for men (with women as the reference group) was consistent over time, ranging from 0.56 to 0.66 in June/July 2021 (study round 13) and February 2021 (study round 9), respectively (online supplemental figure 22). Understanding potential differences in behaviours by demographic group is critical for planning and implementing NPIs.^21^

Our results show that reported behaviours and reasons for leaving the home were strongly correlated with the OxCGRT stringency index and the OxCGRT containment and health index, and the publicly available Google mobility data, demonstrating the potential value of both the Google mobility data and the OxCGRT indices as population-level proxies for social distancing behaviours. These are complementary to individual-based self-reported variables enabling the investigation of potential determinants of behavioural changes throughout the pandemic. However, there is no guarantee these relationships would generalise outside the time periods considered, particularly as signs of pandemic fatigue in later rounds could decrease the correlation between self-reported behaviours and the OxCGRT indices. We also emphasise that our analyses do not indicate whether policy changes are leading or lagging indicators of behavioural change, a key issue in preparing for any future pandemic.

### Limitations

Interpretation of our data is dependent on the accuracy of the self-reported behaviours. Previous studies have indicated that behaviour-related signals may be drowned out by bias and noise in self-report data,^22^ and adherence to protective behaviours may be substantially overestimated in self-reported data both historically^23^ and during the COVID-19 pandemic.^24^ While the strong associations between our data and aggregated community mobility data and policy indices lend some contextual validity to our findings, we cannot exclude biases in reporting, for example, across demographic groups. We made changes to the questionnaire over the nearly 2 years of the study, in some cases to reflect the evolving situation and terminology such as ‘shielding’, ‘support bubbles’ and the tier system of restrictions, which meant that participant responses over time were not always directly comparable. Additionally, the design of the study involving repeated cross-sectional sampling per round meant that the observed trends reflect changes across representative samples of the population, and in this analysis, we did not investigate how an individual’s behaviour changed over time.^1^

## Conclusion

Despite speculation that populations such as England would not be willing and/or able to change their behaviours in response to an infectious disease threat,^25^ our study, along with others, shows that this is patently not the case.^26 27^ We show that the behavioural response closely followed the implementation of government restrictions, but these large behavioural changes may not be replicated in future events, particularly given the strong evidence of ‘pandemic fatigue’ at the end of the study in March 2022. COVID-19 shaped and strengthened many people’s views about the effectiveness of such measures,^28^ and with multiple pandemic-potential infectious diseases being monitored (such as H5N1^29^ and mpox (monkeypox)^30^), understanding people’s behavioural decisions, notably through self-reported data, remains a critical component of future pandemic preparedness.

## Supplementary material

10.1136/bmjph-2025-002851 online supplemental file 1

## Data Availability

Data are available in a public, open access repository. Data are available upon reasonable request.
